# 2-Butoxyethylacetat: MAK-Begründung, Nachtrag

**DOI:** 10.34865/mb11207d11_2ad

**Published:** 2026-06-30

**Authors:** Andrea Hartwig, MAK Commission

**Affiliations:** 1 Institut für Angewandte Biowissenschaften, Abteilung Lebensmittelchemie und Toxikologie, Karlsruher Institut für Technologie (KIT) Adenauerring 20a, Geb. 50.41 76131 Karlsruhe Deutschland; 2 Ständige Senatskommission zur Prüfung gesundheitsschädlicher Arbeitsstoffe, Deutsche Forschungsgemeinschaft, Kennedyallee 40, 53175 Bonn, Deutschland. Weitere Informationen: Ständige Senatskommission zur Prüfung gesundheitsschädlicher Arbeitsstoffe | DFG

**Keywords:** 2-Butoxyethylacetat, Entwicklungstoxizität, Hämatotoxizität, Hämolyse, Reizwirkung, Analogiebetrachtung, erhöhtes Atemvolumen

## Abstract

The German Senate Commission for the Investigation of Health Hazards of Chemical Compounds in the Work Area (MAK Commission) re-evaluated the assignment of 2-butoxyethyl acetate [112-07-2] to Pregnancy Risk Group C (“Damage to the embryo or foetus is unlikely if the MAK value or the BAT value is observed.”). No developmental toxicity studies are available for 2-butoxyethyl acetate. As the substance is rapidly metabolised to acetic acid and 2-butoxyethanol, for which more data are available, 2-butoxyethyl acetate is assessed in analogy. It is assumed that the developmental toxic effects of 2-butoxyethanol in rats are almost exclusively due to the haemolysis caused by its metabolite butoxyacetic acid. Taking into account the increased respiratory volume at the work place and the lower sensitivity of humans compared to rats for the haemolytic effect, the margin between the NOAEC for developmental toxicity and the MAK value of 10 ml/m^3^ is sufficient. Thus, 2-butoxyethanol has been assigned to Pregnancy Risk Group C and by analogy, the assignment to Pregnancy Risk Group C has been confirmed for 2-butoxyethyl acetate at the MAK value of 10 ml/m^3^.

**Table:** 

**MAK-Wert (2007)**	**10 ml/m^3^ (ppm) ≙ 66 mg/m^3^^a)^**
**Spitzenbegrenzung (2007)**	**Kategorie I, Überschreitungsfaktor 2**
	
**Hautresorption (1984)**	**H**
**Sensibilisierende Wirkung**	**–**
**Krebserzeugende Wirkung**	**–**
**Fruchtschädigende Wirkung (1986)**	**Gruppe C**
**Keimzellmutagene Wirkung**	**–**
	
**BAT-Wert (2015)**	**150 mg Butoxyessigsäure (nach Hydrolyse)/g Kreatinin**
CAS-Nr.	112-07-2
Molmasse	160,21 g/mol
Schmelzpunkt	–64,15 °C (ECHA 2022)
Siedepunkt bei 1,01 hPa	192,15 °C (ECHA 2022)
Dichte bei 20 °C	0,9422 g/cm^3^ (ECHA 2022)
Dampfdruck bei 25 °C	0,5 hPa (ECHA 2022)
log K_OW_	1,51 (ECHA 2022)
Löslichkeit	15 000 mg/l Wasser (ECHA 2022)
**1 ml/m^3^ (ppm) ≙ 6,647 mg/m^3^**	**1 mg/m^3^ ≙ 0,150 ml/m^3^ (ppm)**

^a)^ MAK-Wert für die Summe der Luftkonzentrationen von 2-Butoxyethanol und 2-Butoxyethylacetat

Hinweis: Der Stoff kann gleichzeitig als Dampf und Aerosol vorliegen.

Zu 2-Butoxyethylacetat liegen eine Begründung (Henschler 1984), ein Nachtrag zur Spitzenbegrenzung (Greim 2001), eine Gesamtreevaluierung (Greim 2008 a) sowie eine Reevaluierung der kanzerogenen Wirkung (Hartwig und MAK Commission^2020^) vor.

Zu 2‑Butoxyethylacetat sind nur wenige Studien durchgeführt worden. Aus 2-Butoxyethylacetat entsteht im Metabolismus sehr schnell Essigsäure sowie das gut untersuchte 2‑Butoxyethanol. Daher sind für 2‑Butoxyethylacetat die gleichen systemischen Wirkungen wie für 2‑Butoxyethanol zu erwarten (Hartwig und MAK Commission^2020^). Der Blut:Luft-Verteilungskoeffizient, berechnet mit der Formel von Buist et al. (2012) (mit Molmasse 160,21 g/mol, log K_OW_ 1,51, Dampfdruck 0,5 hPa (ECHA 2022)) liegt für 2-Butoxyethylacetat bei 3937. Daher muss das erhöhte Atemvolumen berücksichtigt werden.

Für 2-Butoxyethanol ist eine Reevaluierung der fruchtschädigenden Wirkung vorgenommen worden, da bisher das erhöhte Atemvolumen nicht berücksichtigt worden ist. Daher wird auch für 2‑Butoxyethylacetat ein Nachtrag erstellt.

Studien zur Reproduktionstoxizität liegen für 2-Butoxyethylacetat nicht vor.

## Bewertung

Das grundlegende Vorgehen zur Bewertung eines Arbeitsstoffes ist der MAK- und BAT-Werte-Liste zu entnehmen (DFG 2025).

Kritische Effekte sind wie bei 2-Butoxyethanol die sensorische Reizwirkung nach Inhalation beim Menschen und die hämatotoxische Wirkung bei der Ratte.

**Fruchtschädigende Wirkung. **Zu 2-Butoxyethylacetat liegen keine Studien zur Entwicklungstoxizität vor. Aufgrund der schnellen Metabolisierung von 2-Butoxyethylacetat zu Essigsäure und 2-Butoxyethanol können 2-Butoxyethylacetat die gleichen systemischen Wirkungen wie die von 2-Butoxyethanol unterstellt werden.

2-Butoxyethanol führt zu hämolytischen Effekten, für die Ratten etwa 16-mal empfindlicher sind als Menschen. Der Metabolit Butoxyessigsäure ist verantwortlich für die hämolytischen Wirkungen, 2-Butoxyethanol zeigt keine derartige Aktivität (Hartwig und MAK Commission^2018^, ^2026^).

In pränatalen Entwicklungstoxizitätsstudien an Ratten und Kaninchen führt 2-Butoxyethanol zu entwicklungstoxischen Effekten, wie Ossifikationsverzögerungen und Embryoletalität. Die NOAEC für Entwicklungstoxizität liegt für Ratten bei 50 ml 2-Butoxyethanol/m^3^ und für Kaninchen bei 100 ml 2-Butoxyethanol/m^3^. Unter Berücksichtigung des erhöhten Atemvolumens (1:2) ergeben sich nur 2,5- bzw. 5-fache Abstände zwischen den NOAEC für Entwicklungstoxizität und dem MAK-Wert von 10 ml 2-Butoxyethanol/m^3^. Für das Kaninchen ist eine direkte entwicklungstoxische Wirkung aufgrund der im Vordergrund stehenden Maternaltoxizität, die jedoch nicht durch Hämolyse verursacht wird, nicht anzunehmen.

Es ist plausibel, dass die entwicklungstoxischen Effekte bei der Ratte fast ausschließlich auf die hämolytischen Wirkungen der Butoxyessigsäure zurückzuführen sind, für die der Mensch ungefähr 16-fach weniger empfindlich ist. Wird dieser Speziesunterschied berücksichtigt, kommt es anhand der Daten mit Ratten zu einem ausreichenden Abstand von 40 (2,5 × 16). Damit ist die Zuordnung von 2-Butoxyethanol bei einem MAK-Wert von 10 ml/m^3^ zur Schwangerschaftsgruppe C bestätigt worden (Hartwig und MAK Commission^2026^). Auch Essigsäure ist bei einem MAK-Wert von 10 ml/m^3^ der Schwangerschaftsgruppe C zugeordnet (Greim 2008 b).

Für **2-Butoxyethylacetat** wird in Analogie zu 2-Butoxyethanol die Zuordnung zu Schwangerschaftsgruppe C bei einem MAK-Wert von 10 ml/m^3^ (66 mg/m^3^) bestätigt.
